# Determination of Glucocorticoids in UPLC-MS in Environmental Samples from an Occupational Setting

**DOI:** 10.1155/2015/678579

**Published:** 2015-03-03

**Authors:** Enrico Oddone, Sara Negri, Massimo Bellinzona, Silvia Martino, Marcello Di Tuccio, Elena Grignani, Danilo Cottica, Marcello Imbriani

**Affiliations:** ^1^Department of Public Health, Experimental and Forensic Medicine, University of Pavia, 27100 Pavia, Italy; ^2^Environmental Research Center, Salvatore Maugeri Foundation, Via S. Maugeri 10, 27100 Pavia, Italy; ^3^Hospital Operating Unit of Occupational Medicine (UOOML), Salvatore Maugeri Foundation, Via S. Maugeri 10, 27100 Pavia, Italy

## Abstract

Occupational exposures to glucocorticoids are still a neglected issue in some work environments, including pharmaceutical plants. We developed an analytical method to quantify simultaneously 21 glucocorticoids using UPLC coupled with mass spectrometry to provide a basis to carry out environmental monitoring. Samples were taken from air, hand-washing tests, pad-tests and wipe-tests. This paper reports the contents of the analytical methodology, along with the results of this extensive environmental and personal monitoring of glucocorticoids. The method in UPLC-MS turned out to be suitable and effective for the aim of the study. Wipe-test and pad-test desorption was carried out using 50 mL syringes, a simple technique that saves time without adversely affecting analyte recovery. Results showed a widespread environmental pollution due to glucocorticoids. This is of particular concern. Evaluation of the dose absorbed by each worker and identification of a biomarker for occupational exposure will contribute to assessment and prevention of occupational exposure.

## 1. Introduction

Under physiological conditions, glucocorticoids possess various functions including modulation of carbohydrate, protein, lipid, and nucleic acid metabolism; sharp increases are observed in response to stressors that threaten homeostasis. These properties, and especially the anti-inflammatory activity, are extensively used in pharmaceutical industry to produce several medical formulations.

This industrial manufacturing could lead to some exposures to glucocorticoids in occupational settings that possibly entail hazards for workers' health, thus far not adequately considered nor thoroughly investigated.

Some evidence [1–3] pointed out Cushingoid symptoms, adrenocortical insufficiency, and skin disorders due to chronic accidental absorption of glucocorticoids. To our knowledge, from early 80's to date no other study focused on this specific issue and therefore no assessment of environmental monitoring or human biomonitoring was provided despite the wide variety of clinical problems related to therapies with glucocorticoids and the low plasmatic level at which these molecules execute their endocrine action [4].

Furthermore, occupational exposures generally entail low doses of environmental pollutants assumed for long periods. The effect of such a kind of exposure to glucocorticoids is thus far neglected as well as the simultaneous exposure to different molecules.

Such occupational exposures start to concern occupational physicians and industrial hygienists, requiring the development of analytical techniques is accurate and reliable.

In this regard, our survey needed the measurement of 21 different glucocorticoids from air, hand washing, pad-test, and wipe-test (UNI CEN/TS 15279:2006). Thus, an adequate analytical method to quantify simultaneously 21 different compounds of interest using UPLC and tandem mass spectrometry was developed.

This paper reports the contents of the analytical methodology, along with the results of this extensive environmental and personal monitoring of glucocorticoids, carried out in a pharmaceutical industry plant.

## 2. Materials and Methods

### 2.1. General Setting

The industrial plant, in which the monitoring was carried out, produces several active pharmaceutical compounds, mainly steroids, antibiotics, and anticancer drugs. Annually, about 100 tons of pharmacologically active raw materials are used in the plant.

The production is generally made of batch processes, related to the industrial orders, and is carried out in closed-loop systems. Each class of drugs is produced in a specific department of the plant, distinct from the others.

The glucocorticoids synthesis is made up by several productive steps, namely, the washout of the reactor with water and subsequently with acetone and/or methanol; inerting and anhydrification; loading of the raw materials; specific chemical reactions; precipitation or crystallization of the products; centrifugation; drying; packaging.

Among those steps, only the drying could expose workers to glucocorticoids, given that the pharmacologically active compounds are manually introduced in the drying ovens and manually extracted from them. In fact, the operators, after cleaning their workplace with a pressure washer and acetone, have to arrange the semifinished products on trays and place them in the drying ovens. After an appropriate time lapse, depending on the glucocorticoids, workers have to extract trays and to stock the products in specific kegs. Finally, the personnel have to clean again their workplace with a pressure washer and acetone.

To effectively monitor this working environment and workers' exposures, samples of air, hand-washing test, pad-test, and wipe-test were collected to assess the contamination of work surfaces and work clothing, as described as follows. A list of glucocorticoids considered in this study is provided in Table 1.

### 2.2. Samples Collection

All samples were collected during 5 days in November 2013, in which beclomethasone dipropionate, mometasone furoate, prednisolone 17-valerate, and 21-acetate were produced.

Air samples were collected in workers' breathing zone by means of a pump which draws air through a 25 mm diameter fiberglass filter at a flow rate of 1.4 L/min. For each shift, an operator was monitored.

Moreover, workers' personal exposures were assessed with hand-washing test. This sampling technique consists in hand washing with an appropriate solvent at the end of the work shift and after that gloves have been removed. In particular, 250 mL of 50% ethanol solution have been directly poured on the operators' hands and recollected in a specific container, after some rubbings (“pouring method”) [5–7]. The ethanol has been chosen as solvent given its low skin toxicity and its capability to remove even small amounts of not solubilized pollutants by mechanical action. Sampling has been carried out both after the introduction of semifinished products in drying ovens and after the extraction from drying ovens.

Contamination of work clothes was assessed by means of four pad-tests (gauzes in TNT, 10 × 10 cm). They were clamped on the workers' smock external surface at thorax, back, right forearm, left forearm, right thigh, and left thigh. In addition, a pad-test was fixed on the internal surface of the workers' smock at right forearm and left forearm [8]. At the end of the work shift samples have been removed from the smock, sealed in syringes, and conserved at 4°C until the day of the analysis.

The assessment of work surfaces' contamination has been carried out with the wipe-test, in which the sampling substratum was provided by four-layer nonwoven wipes of 10 × 10 cm dampened with 2 mL of ethanol-water solution (50 : 50, v/v). The sampling was carried out by wiping all across the selected surfaces, namely, the worktop next to the extractor (20 × 20 cm, 1 sample), the door stops (60 × 15 cm, 8 samples), the door handles (5 samples), the keg cover (20 × 20 cm, 1 sample) and the keg lateral surface (20 × 20 cm, 1 sample). As for the pads, wipes were sealed in syringes and conserved at 4°C.

### 2.3. Determination of Glucocorticoids in UPLC-MS

Standards of the analytes of interest were provided by the producing company with a degree of purity >95%, while the carbamazepine internal standard (degree of purity >98%), the methanol for the LC-MS instrument, and the formic acid (HiPerSolv degree of purity) were provided by Sigma Aldrich (Munich, Germany). Moreover, water for the mobile phase was daily supplied by a MilliporeMilliQ purifier and for the samples' desorption, solvents with purity AnalaR were used.

The determination of glucocorticoids in wipe-, pad-, and hand-washing tests and air samples was obtained by liquid chromatography associated with mass spectrometry, namely, with Acquity UPLC system coupled with a triple quadrupole Waters TQD mass spectrometer (Waters, Milford, MA, USA). The Mass Lynx 4.1 software oversaw the instrument and TargetLynx software was used for quantification.

Briefly, after sampling, wipe- and pad-test were desorbed using a 50 mL syringe with 5 mL × 3 of methanol-acetonitrile (50 : 50, v/v), centrifuged, conveniently diluted before injection in UPLC (range of calibration curve: 10–100 *μ*g); hand washing was realized with 250 mL ethanol-water solution (50 : 50, v/v); then an aliquot was simply centrifuged and diluted before injection (range of calibration curve: 0.17–1.75 mg); fiber glass membrane was desorbed with 2 mL of methanol-acetonitrile (50 : 50, v/v), centrifuged, and diluted before injection (range of calibration curve: 1.4–14 ng).

Chromatographic separation was performed on a UPLC HSS C18 column (2.1 × 50 mm, 1.8 *μ*m) maintained at 30°C and by gradient elution with a mixture containing variable proportions of 0.1% formic acid solution and methanol delivered at the flow rate of 0.5 mL/min. The gradient program was 50% methanol for 0.5 min; was from 50% to 98% methanol in 9.5 min (linear gradient) and was held for 2.5 min to permit the washing of the column; was from 98% to 50% methanol in 1 min (linear gradient) and was held for 1.5 min (column reconditioning); retention time of carbamazepine (internal standard) and other analytes are provided in Table 1.

**Figure 1:**
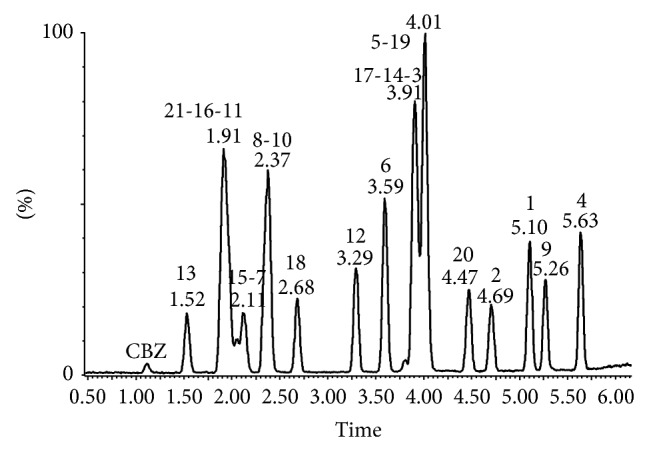
Example of chromatographic separation related to a single point of calibration curve (50 *μ*g on wipe-test). Numbers on peaks identify glucocorticoids code as showed in Table 1.

For the detection of the 21 glucocorticoids in mass spectrometry, electrospray was operated in positive ion mode and the acquisition was performed in single ion recording (SIR). An example of chromatographic separation is reported in Figure 1. The correspondence between the number of the peaks and the analytes is indicated in Table 1.

## 3. Results

The method in UPLC-MS developed for the determination of 21 glucocorticoids simultaneously was suitable and effective for the aim of the study.

Limits of detection (calculated as signal-to-noise ratio of 3) of different analytes were within the range of 0.1–1.4 *μ*g for wipe-test and pad-tests, 2–23 *μ*g for hand-washing tests, and 0.25–0.52 *μ*g/m^3^ for air samples, considering a sampling average time of two hours.

Tables 2 and 3 show results of the determinations in all kinds of samples. These results account for the viability of this method to assess the workers' exposure.

The environmental pollution due to glucocorticoids seemed to be widespread. The work surfaces and environment were contaminated with several different analytes, whose mean values often exceeded 10 *μ*g and not infrequently overtook 100 *μ*g. Moreover, the maximum value in wipe-test determinations exceeded 1 mg in two cases: beclomethasone dipropionate (3.37 mg, during the day of compound production) and chlormadinone acetate (1.22 mg) (Table 2).

Air monitoring showed that during the days in which glucocorticoids were processed, the compound in production held the highest value. Beclomethasone dipropionate showed the maximum concentration, reaching 388.2 *μ*g/m^3^ (Table 2). It is interesting to note that chlormadinone acetate and mometasone furoate reached the concentration of 1.28 *μ*g/m^3^ and 1.22 *μ*g/m^3^, respectively, also in a day in which the production was devoted to beclomethasone dipropionate (Table 2).

The majority of mean values of the hand-washing test were above 10 *μ*g, rising to 220.2 *μ*g and 258.4 *μ*g for desoximetasone and beclomethasone dipropionate, respectively. Monitored workers were occasionally exposed to nearly or over 1 mg of glucocorticoids (0.83 mg and 2.04 mg for beclomethasone dipropionate and desoximetasone, resp.) (Table 2).

Considering pad-tests on the external surface of the workers' smock, results were especially high for beclomethasone dipropionate, mometasone furoate, and prednisolone 17-valerate 21-acetate, as expected given that they were the compound in production. The mean values of these analytes were almost constantly over 10 *μ*g, rising to 0.13–0.66 mg for beclomethasone dipropionate and mometasone furoate. As expected, minimum values were found for pad-tests clamped on the back of workers' smock. These results are consistent with values from pad-tests placed on the internal surface of the smock, at right and left forearms, although, for these pad-tests, not entirely negligible values were found also for chlormadinone acetate and desoximetasone. Internal pad-tests showed mean values 50–100-fold lower than external one (Table 3).

## 4. Discussion

Our study shows results from an occupational environment monitoring that challenged our laboratory to develop a method to simultaneously quantify in UPLC-MS 21 different glucocorticoids from wipe-tests, pad-tests, hand washing, and fiberglass filters. To our knowledge, in the literature there are no papers that report methods to quantify these analytes with this chromatographic technique in such substrates. In the first chromatographic tests we took into consideration the chromatographic conditions (type of column and mobile phase) reported by some authors [9, 10], although these works deal with determinations of few glucocorticoids in biological matrices, such as urine or hair, for clinical purposes.

Usually, in the first development phases of an LC-MS method it is essential to consider both the target regarding the range of calibration curve and limit of quantification. These parameters depend on the real concentration of samples. Unfortunately, we had no such literature information and thus it was necessary to make some proofs directly on industrial samples. It was immediately clear that samples were characterized by a high between-samples and between-analytes variability and so the calibration curve range was chosen in such a manner as to avoid signal saturation. Samples with a signal out from this range were opportunely diluted and injected again.

Initially, carbamazepine (CBZ) was chosen as internal standard given that this compound was not present in the work environment and its hydrophobicity is similar to glucocorticoids. Nevertheless, after the first analyses on real samples it was decided to add CBZ to all samples although its use was avoided for quantifications, due to high glucocorticoids signal variability. It has not been possible to define a dilution factor before first injection in UPLC and, accordingly, the right initial internal standard spike. In addition, to control for possible instrument drift and quantitative performance issues an external standard was regularly injected every 20 samples.

Due to time constraints described below and the lack of available guidelines, we could not carry out a formal method validation; nevertheless, we verified the method's reliability as described in the following.

Recovery of the analytes and matrix effect were verified for each kind of sample by calculating the ratio of the peak area in the presence of matrix (wipe-test, hand washing, or membrane) to the peak area in the absence of matrix (pure solution of the analytes diluted in the same mobile phase). This test was carried out for low and high level concentrations. For wipe-test we confirmed this procedure by spiking standards after cleaning different types of surfaces not contaminated by glucocorticoids; similarly, for hand washing we carried out the test using samples taken from subjects not exposed to our analytes.

Moreover, this survey was planned according to company demand since some cases of fingers' telangiectasia have been observed among workers. Thus, given that results were waited within a brief time, to shorten the method development without threatening the quality of result, we decided not to infuse standards into the mass spectrometer to obtain transitions of each molecule (MRM). After some injections of each single analyte at different cone voltage, we decided to quantify the analytes in SIM (Table 1). We thought that the choice of reducing the method's selectivity did not penalize the analysis of environmental samples, generally cleaner and ore freer from analytical interferences than biological samples. In fact, the analyses carried out on real blank samples (i.e., wipe-test simply obtained after cleaning different types of surfaces or hand washing from subjects not exposed to glucocorticoids) did not show chromatographic interferences with the same RT and the same* m/z*.

Besides, the high level of dilution step of the samples necessary to quantify the analytes within the range of calibration curve contributed to obtain samples particularly clean and thus free from interferences.

Wipe-tests and pad-tests desorption was carried out by using syringes 50 mL, an innovative technique that saves time without affecting the analyte recovery. No difference was observed between wipe- and pad-tests regarding analyte recovery: results were encouraging showing a recovery mean for all analytes of 92.4%. Indeed, the desorption of this kind of samples is usually carried out by submerging the gauze into a container 100 mL with a desorption volume of 20–25 mL (e.g., urine container) and leaving the sample plunged and shacked for at least 30 minutes [11, 12]. Our method requires a desorption volume of 15 mL only, allowing desorption of samples in a few minutes. Furthermore, it was not necessary to introduce a purification step (typically solid phase extraction) because samples were clean enough and concentrated; on the contrary, after desorption, many samples were opportunely diluted and injected in a second time to return to the range of instrument linearity.

Our survey shows a wide contamination of occupational environment, both for work surfaces and for structures. Also air samples highlight a respiratory exposure for some glucocorticoids (in particular, beclomethasone dipropionate, i.e., the plant most produced glucocorticoid). The smocks are probably a good protective device, although samples on their internal surface at forearms are not negligible for some compounds (Table 3). This is an indirect proof of a skin contact, as well as the results from hand-washing test that underline a certain exposure although workers wear their protective gloves. Nevertheless, results from hand-washing tests represent the most relevant contamination issue from an occupational health point of view. In fact, these workers are daily exposed to an average dose of about 0.8 mg, considering all glucocorticoids. Some of them (beclomethasone dipropionate, desoximetasone) showed single maximum results close to 1 mg (Table 2). For example, assuming that a single application of a beclomethasone cream (0.1%) to hands is about 1 gram, the dose measured on workers' hands is close to the pharmacological one. Furthermore, the effect of multiple contemporary exposures cannot be disregarded, especially when synergic and addictive effects were taken into account.

In addition, the widespread contamination of work environment and surfaces posits a cross contamination issue of the pharmacological product that has to be dealt with.

Therefore, we think that environmental monitoring of exposed workers should no longer be neglected and our analytic method provides a basis to develop further surveys for measuring and limiting the exposures.

## 5. Conclusions

Our development of an analytical method to simultaneously quantify 21 different glucocorticoids seemed to be effective and reliable, along with the innovative desorption technique using 50 mL syringes. These methods allow us to carry out an extensive (environmental and personal) monitoring in a pharmaceutical plant, showing a widespread contamination that should no longer be neglected. Moreover, to evaluate the real dose absorbed by each worker, it is of particular concern to identify an effective biomarker of occupational exposure.

## Figures and Tables

**Table 1:** Codes and principal chemical features of glucocorticoids.

Compound	Code	Case number	RT^a^	*m*/*z*	CV^b^
Beclomethasone dipropionate	1	5534-09-8	5.10	522.0	36
Betamethasone dipropionate	2	5593-20-4	4.69	505.6	35
Clobetasol 17-propionate	3	25122-46-7	3.49	468.0	30
Clocortolone pivalate	4	34097-16-0	5.63	496.0	30
Chlormadinone acetate	5	302-22-7	4.00	405.9	32
Delmadinone acetate	6	13698-48-2	3.59	403.9	25
Desonide	7	638-94-8	2.12	417.5	30
Desoximetasone	8	382-67-2	2.35	377.5	30
Diflucortolone valerate	9	59198-70-8	5.26	479.6	30
Exemestane	10	107868-30-4	2.38	297.4	35
Fluocinolone acetonide	11	67-73-2	1.96	453.5	35
Fluocinonide	12	356-12-7	3.29	495.5	30
Fluprednidene acetate	13	1255-35-2	1.52	433.5	30
Halcinonide	14	3093-35-4	3.92	456.0	20
Halometasone monohydrate	15	50629-82-8	2.04	445.9	19
Hydrocortisone acetate	16	50-03-3	1.92	405.5	40
Megestrol acetate	17	595-33-5	3.89	385.5	20
Methylprednisolone acetate	18	53-36-1	2.67	339.2	40
Mometasone furoate	19	83919-23-7	4.03	522.4	30
Prednisolone 17-valerate 21-acetate	20	72064-79-0	4.47	487.6	30
Triamcinolone acetonide	21	76-25-5	1.88	435.5	25
Carbamazepine^c^	CBZ	298-46-4	1.10	237.3	45

^a^Retention time.

^b^Cone Voltage.

^c^Internal standard.

**Table 2:** Results of environmental and personal monitoring of glucocorticoids in a pharmaceutical plant (2013).

Compound	Door handles^a^			Door stops^a^			Air samples^b^			Hand washing^a^		
	(*N* = 8)			(*N* = 5)			(*N* = 5)			(*N* = 10)		
	Min	Max	Mean [Std]	Min	Max	Mean [Std]	Min	Max	Mean [Std]	Min	Max	Mean [Std]
Beclomethasone dipropionate	99.8	413.3	***252.7*** [145.1]	193.3	3368.1	***910.0*** [1377.7]	1.8	388.2	***87.2*** [168.4]	26.8	833.8	***258.4*** [328.8]

Betamethasone dipropionate	17.8	89.9	***48.9*** [26.8]	23.8	168.0	***90.8*** [56.9]	<0.1	<0.1	***<0.1*** [—]	4.0	13.0	***7.9*** [3.5]

Clobetasol 17-propionate	54.3	104.2	***69.0*** [29.2]	21.7	481.0	***160.2*** [186.0]	<0.1	0.9	***0.2*** [0.4]	11.8	33.0	***19.8*** [8.2]

Clocortolone pivalate	1.9	30.4	***12.1*** [11.2]	1.7	77.7	***26.4*** [30.8]	<0.1	<0.1	***<0.1*** [—]	8.0	8.0	***8.0*** [—]

Chlormadinone acetate	8.0	64.8	***32.0*** [21.9]	8.9	1219.6	***298.0*** [21.9]	<0.1	1.3	***0.4*** [0.5]	5.4	126.0	***66.4*** [54.4]

Delmadinone acetate	<0.1	2.0	***0.4*** [0.9]	<0.1	37.6	***8.0*** [16.5]	<0.1	<0.1	***<0.1*** [—]	10.0	10.0	***10.0*** [—]

Desonide	3.8	43.6	***16.3*** [15.8]	4.5	44.0	***20.6*** [15.3]	<0.1	<0.1	***<0.1*** [—]	4.4	74.4	***26.9*** [29.8]

Desoximetasone	2.4	16.5	***10.0*** [5.5]	2.6	50.2	***25.5*** [19.2]	<0.1	2.0	***<0.4*** [0.9]	0.8	1074.3	***220.2*** [477.5]

Diflucortolone valerate	2.9	36.3	***13.6*** [13.3]	4.7	90.9	***38.6*** [32.3]	<0.1	<0.1	***<0.1*** [—]	4.0	53.8	***14.5*** [22.0]

Exemestane	<0.1	1.3	***0.4*** [0.6]	<0.1	41.5	***8.6*** [18.4]	<0.1	<0.1	***<0.1*** [—]	9.0	9.0	***9.0*** [—]

Fluocinolone acetonide	6.7	52.1	***23.2*** [17.8]	5.3	47.5	***24.1*** [18.0]	<0.1	<0.1	***<0.1*** [—]	5.0	30.0	***17.0*** [11.1]

Fluocinonide	17.2	73.4	***42.8*** [21.2]	16.5	109.4	***58.9*** [39.1]	<0.1	0.7	***0.2*** [0.3]	7.0	59.5	***26.8*** [21.7]

Fluprednidene acetate	5.9	35.2	***13.7*** [12.2]	5.3	79.6	***32.2*** [28.1]	<0.1	<0.1	***<0.1*** [—]	5.0	5.4	***5.1*** [0.2]

Halcinonide	<0.1	0.5	***0.2*** [0.3]	<0.1	1.2	***0.5*** [0.4]	<0.1	<0.1	***<0.1*** [—]	3.8	4.0	***4.0*** [0.1]

Halometasone monohydrate	1.0	7.8	***4.3*** [2.9]	1.0	13.1	***7.1*** [4.8]	<0.1	<0.1	***<0.1*** [—]	13.0	13.0	***13.0*** [—]

Hydrocortisone acetate	3.2	32.8	***14.6*** [11.9]	3.5	115.9	***38.0*** [46.2]	<0.1	<0.1	***<0.1*** [—]	2.0	2.1	***2.0*** [0.1]

Megestrol acetate	3.1	31.5	***9.5*** [12.3]	5.3	86.6	***26.8*** [33.7]	<0.1	1.7	***0.4*** [0.8]	5.4	86.0	***22.4*** [35.6]

Methylprednisolone acetate	<0.1	1.4	***0.6*** [0.7]	<0.1	3.0	***1.4*** [1.4]	<0.1	<0.1	***<0.1*** [—]	23.0	23.0	***23.0*** [—]

Mometasone furoate	7.6	270.0	***93.8*** [109.5]	15.4	182.9	***60.9*** [70.4]	<0.1	58.3	***12.0*** [25.9]	12.0	128.8	***58.6*** [47.7]

Prednisolone 17-valerate 21-acetate	4.4	17.1	***9.3*** [5.2]	1.6	22.7	***10.7*** [7.7]	<0.1	21.0	***4.2*** [9.4]	6.0	23.9	***9.6*** [8.0]

Triamcinolone acetonide	10.8	63.5	***30.5*** [22.5]	18.8	92.1	***49.9*** [30.7]	<0.1	<0.1	***<0.1*** [—]	4.9	13.1	***6.8*** [3.6]

^a^Absolute *µ*g.

^b^ *µ*g/m^3^.

**Table 3:** Results of pad-tests on workers clothes of a pharmaceutical plant (2013). For each sample *N* = 5. “I” indicates the internal surface of the smocks.

Compound	Torax Mean (Min–Max) [Std]	Right forearm Mean (Min–Max) [Std]	Left forearm Mean (Min–Max) [Std]	Back Mean (Min–Max) [Std]	Right thigh Mean (Min–Max) [Std]	Left thigh Mean (Min–Max) [Std]	Right forearm - I Mean (Min–Max) [Std]	Left forearm - I Mean (Min–Max) [Std]
Beclomethasone dipropionate	**661.7** (5.5–3203.4) [1421.0]	**253.0** (6.1–1095.7) [472.0]	**473.2** (7.8–2254.2) [995.7]	**11.0** (0.7–48.8) [21.2]	**294.0** (17.3–1138.0) [479.4]	**298.3** (1.0–1080.2) [466.7]	**9.4** (0.8–19.1) [6.66]	**8.4** (3.9–14.8) [4.60]

Betamethasone dipropionate	**2.4** (0–4.1) [1.9]	**3.0** (0–5.1) [2.0]	**3.7** (1.9–4.6) [1.1]	**0.1** (0–0.4) [0.2]	**4.7** (0.8–9.8) [3.3]	**1.4** (0.7–2.8) [0.9]	**0.4** (0–1.1) [0.4]	**0.8** (0–2.2) [0.9]

Clobetasol 17-propionate	**2.7** (1.4–4.0) [1.3]	**4.1** (1.6–7.5) [2.4]	**3.5** (1.9–6.0) [1.7]	**0.3** (0–0.5) [0.3]	**10.8** (3.3–31.0) [11.4]	**6.3** (1.0–25.8) [10.9]	**1.9** (0.5–4.9) [1.7]	**1.2** (0.5–2.6) [0.9]

Clocortolone pivalate	**0.1** (0–0.5) [0.2]	**0.2** (0–0.5) [0.3]	**0.1 **(0–0.5) [0.2]	**0.1** (0–0.5) [0.2]	**0.4** (0–0.5) [0.2]	**0.1** (0–0.5) [0.2]	**0.1** (0–0.5) [0.2]	**0.1** (0–0.5) [0.2]

Chlormadinone acetate	**6.2** (1.5–14.9) [6.2]	**5.5** (2.3–10.0) [3.2]	**6.2** (1.0–16.2) [5.8]	**0.7** (0–1.1) [0.5]	**8.3** (2.0–18.6) [7.1]	**3.8** (0.5–7.5) [2.6]	**10.0** (0–25.0) [11.1]	**5.8** (3.9–8.2) [2.1]

Delmadinone acetate	**<0.1** (—) [—]	**<0.1** (—) [—]	**<0.1** (—) [—]	**<0.1** (—) [—]	**<0.1** (—) [—]	**<0.1** (—) [—]	**<0.1** (—) [—]	**<0.1** (—) [—]

Desonide	**4.1** (0–16.6) [7.0]	**5.5** (0–21.7) [9.2]	**4.3** (0–14.7) [6.0]	**<0.1** (—) [—]	**5.4** (0.8–18.9) [7.7]	**2.2** (0–6.9) [2.9]	**1.5** (0–3.7) [1.8]	**0.8** (0–3.4) [1.5]

Desoximetasone	**1.9** (0–5.1) [2.4]	**2.4** (0–8.1) [3.9]	**6.9** (0–26.7) [13.2]	**<0.1** (—) [—]	**3.3** (0–10.9) [5.2]	**2.6** (0–10.6) [5.3]	**5.7** (0–22.8) [11.4]	**3.3** (0–12.4) [6.1]

Diflucortolone valerate	**0.5** (0–1.1) [0.5]	**0.8** (0–1.4) [0.5]	**1.2** (0–4.1) [1.7]	**<0.1** (—) [—]	**0.9** (0–2.1) [1.0]	**0.4** (0–1.9) [0.8]	**3.4** (0–17.2) [7.7]	**7.7** (0–30.2) [15.0]

Exemestane	**<0.1** (—) [—]	**<0.1** (—) [—]	**<0.1 ** (—) [—]	**<0.1** (—) [—]	**<0.1** (—) [—]	**<0.1** (—) [—]	**<0.1** (—) [—]	**<0.1** (—) [—]

Fluocinolone acetonide	**1.1** (0–1.9) [0.7]	**2.6** (0–9.5) [3.9]	**2.6** (0–10.6) [4.5]	**<0.1** (—) [—]	**3.2** (0–10.8) [4.3]	**1.6** (0–6.2) [2.7]	**0.5** (0–1.7) [0.8]	**0.8** (0–2.5) [1.2]

Fluocinonide	**2.8** (0.5–6.3) [2.7]	**7.8** (1.2–26.5) [10.6]	**7.7** (0.8–30.2) [12.7]	**<0.1** (—) [—]	**6.5** (1.4–19.0) [7.4]	**3.9** (0–16.6) [7.1]	**2.0** (0–6.0) [2.5]	**1.5** (0–4.6) [1.9]

Fluprednidene acetate	**0.3** (0–0.9) [0.4]	**0.5** (0–0.8) [0.3]	**0.8** (0–3.0) [1.2]	**<0.1** (—) [—]	**0.9** (0–1.7) [0.7]	**<0.1** (—) [—]	**<0.1** (—) [—]	**<0.1** (—) [—]

Halcinonide	**<0.1** (—) [—]	**<0.1** (—) [—]	**<0.1** (—) [—]	**<0.1** (—) [—]	**<0.1** (—) [—]	**<0.1** (—) [—]	**<0.1** (—) [—]	**<0.1** (—) [—]

Halometasone monohydrate	**1.0** (1.0–1.1) [0]	**1.4** (1.0–2.8) [0.8]	**1.2** (1.0–2.1) [0.5]	**1.0** 1.0 [0]	**1.5** (1.0–3.3) [1.0]	**1.0** 1.0 [0]	**1.0** 1.0 [0]	**1.0** 1.0 [0]

Hydrocortisone acetate	**<0.1** (—) [—]	**<0.1** (—) [—]	**<0.1** (—) [—]	**<0.1** (—) [—]	**<0.1** (—) [—]	**<0.1** (—) [—]	**<0.1** (—) [—]	**<0.1** (—) [—]

Megestrol acetate	**6.9** (0–34.0) [15.1]	**2.1** (0–8.0) [3.4]	**8.3** (0–40.0) [17.7]	**0.8** (0–3.0) [1.5]	**15.1** (0.5–73.0) [32.3]	**1.5** (0–7.0) [3.1]	**1.9** (0–8.0) [3.5]	**2.1** (0–9.0) [3.9]

Methylprednisolone acetate	**1.4** 1.4 [0]	**1.4** 1.4 [0]	**1.4** 1.4 [0]	**1.4** 1.4 [0]	**1.4** 1.4 [0]	**1.4** 1.4 [0]	**1.4** 1.4 [0]	**1.4** 1.4 [0]

Mometasone furoate	**129.3** (8.8–453.1) [216.4]	**418.3** (2.8–1573.0) [770.9]	**576.2** (7.2–2262.6) [1124.3]	**10.9** (0–42.0) [20.7]	**414.0** (8.7–1554.4) [760.6]	**372.2** (2.0–1445.7) [715.8]	**10.6** (2.0–20.8) [9.4]	**9.5** (1.5–31.0) [14.4]

Prednisolone 17-valerate 21-acetate	**37.9** (0–187.7) [83.8]	**93.8** (0–466.8) [208.5]	**44.0** (0–218.8) [97.7]	**0.9** (0–4.7) [2.1]	**61.9** (0–305.9) [136.4]	**10.2** (0–50.3) [22.4]	**1.6** (0–8.0) [3.6]	**0.6** (0–3.0) [1.3]

Triamcinolone acetonide	**2.5** (0–8.4) [3.6]	**3.5** (0–12.6) [5.2]	**2.2** (0.3–5.2) [2.0]	**<0.1** (—) [—]	**3.4** (0–9.1) [3.7]	**0.6** (0–1.8) [0.7]	**0.2** (0–0.9) [0.4]	**0.4** (0–1.4) [0.6]

## References

[1] Farina G., de Micheli P., Basso P., Castelli P. P., Locati G., Secchi G. C. (1977). Technopathy from steroids in the drug industry: first report of a syndrome from exogenous hypercortisolism in workers engaged in the production of steroids. *Medicina del Lavoro*.

[2] Newton R. W., Browning M. C. K., Iqbal J., Piercy N., Adamson D. G. (1978). Adrenocortical suppression in workers manufacturing synthetic glucocorticoids. *British Medical Journal*.

[3] Newton R. W., Browning M. C. K., Nicholson P. C., Mowat D. A. E. (1982). Adrenocortical suppression in workers employed in manufacturing synthetic glucocorticosteroids: solutions to a problem. *British Journal of Industrial Medicine*.

[4] Vandenberg L. N., Colborn T., Hayes T. B. (2012). Hormones and endocrine-disrupting chemicals: low-dose effects and nonmonotonic dose responses. *Endocrine Reviews*.

[5] Fenske R. A., Schulter C., Lu C., Allen E. H. (1998). Incomplete removal of the pesticide captan from skin by standard handwash exposure assessment procedures. *Bulletin of Environmental Contamination and Toxicology*.

[6] Brouwer D. H., Boeniger M. F., van Hemmen J. (2000). Hand wash and manual skin wipes. *The Annals of Occupational Hygiene*.

[7] Marquart H., Brouwer D. H., van Hemmen J. J. (2002). Removing pesticides from the hands with a simple washing procedure using soap and water. *Journal of Occupational and Environmental Medicine*.

[8] Aprea C., Sciarra C., Lunghini L., Bozzi N., Minoia L. (2000). Pesticides. *Environmental and Biological Monitoring of Occupational Exposures to Xenobiotics*.

[9] Polettini A., Bouland G. M., Montagna M. (1998). Development of a coupled-column liquid chromatographic-tandem mass spectrometric method for the direct determination of betamethasone in urine. *Journal of Chromatography B: Biomedical Applications*.

[10] Cirimele V., Kintz P., Dumestre V., Goullé J. P., Ludes B. (2000). Identification of ten corticosteroids in human hair by liquid chromatography-ionspray mass spectrometry. *Forensic Science International*.

[11] Hedmer M., Jönsson B. A. G., Nygren O. (2004). Development and validation of methods for environmental monitoring of cyclophosphamide in workplaces. *Journal of Environmental Monitoring*.

[12] Sottani C., Turci R., Schierl R. (2007). Simultaneous determination of gemcitabine, taxol, cyclophosphamide and ifosfamide in wipe samples by high-performance liquid chromatography/tandem mass spectrometry: protocol of validation and uncertainty of measurement. *Rapid Communications in Mass Spectrometry*.

